# Endoscopic ultrasound-guided ileocolonic anastomosis for relief malignant bowel obstruction

**DOI:** 10.1055/a-2749-7754

**Published:** 2025-12-15

**Authors:** Haiping Peng, Qingyan Fu, Tian Zheng, Tuo Zhou, Da Li, Xiang Ding

**Affiliations:** 1 658843 Department of Gastroenterology, Yueyang Central Hospital, Yueyang, China; 2 658843 Department of Medical Imaging, Yueyang Central Hospital, Yueyang, China

A 71-year-old patient was referred for 13-year recurrent cough/expectoration, 10-year post-exertional dyspnea, and 1-day worsening abdominal pain/distension. He had recurrent acute exacerbations of chronic obstructive pulmonary disease , left lung squamous cell carcinoma (Stage IB), and coronary heart disease. Computed tomography (CT) showed a distal ileal mass (≈5th group), luminal narrowing, and proximal small bowel obstruction ( Fig. 1 ). Surgery was suggested but declined by the family due to poor pulmonary function and high risk. Endoscopic ultrasound-guided ileocolonic anastomosis (EUS-ICA) under general anesthesia was performed after multidisciplinary discussion ( Video 1 ).

**Fig. 1:**
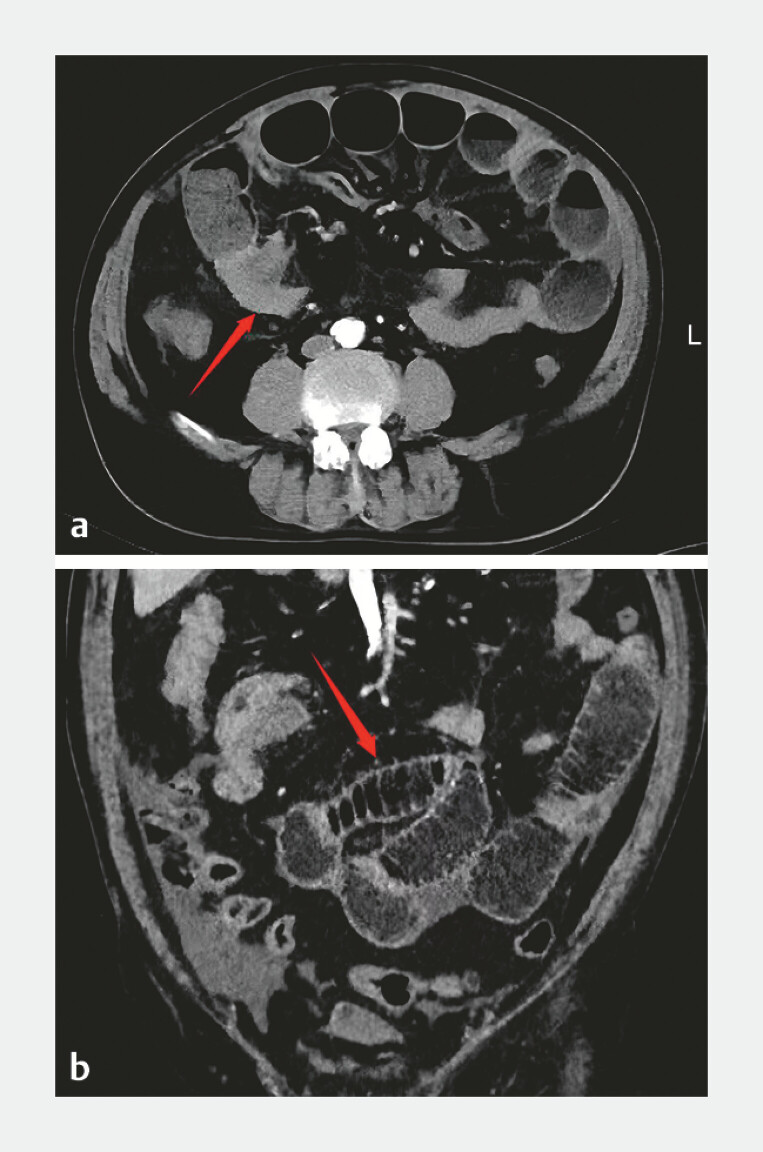
**a** Preoperative axial CT: distal ileal mass (fifth group,) with luminal narrowing and proximal small bowel obstruction. **b** Preoperative coronal CT: distal ileal mass (fifth group,) with luminal narrowing and proximal small bowel obstruction. CT, computed tomography.

Detailed operation of EUS-guided ileocolonic anastomosis combined with LAMS placement for MBO. MBO, Malignant bowel obstruction.Video 1

Postoperatively, the patient passed stool the same day; follow-up CT showed improved small bowel dilatation ( Fig. 2 ). On postoperative day 2, stent balloon dilation ( Fig. 3 ) relieved abdominal distension and restored defecation/flatulence. He was transitioned to oral nutrition, discharged on day 4, and remained well on follow-up.

**Fig. 2:**
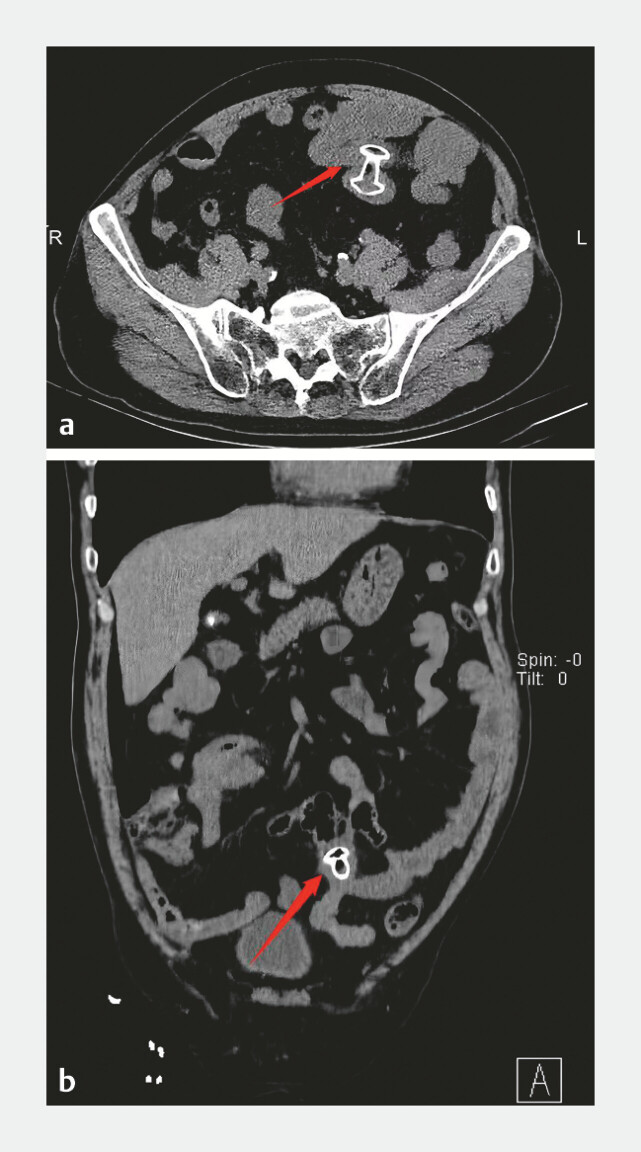
**a** Postoperative axial CT revealed intestinal dilatation, with the stent in place. **b** Postoperative coronal CT revealed intestinal dilatation, with the stent in place. CT, computed tomography.

**Fig. 3:**
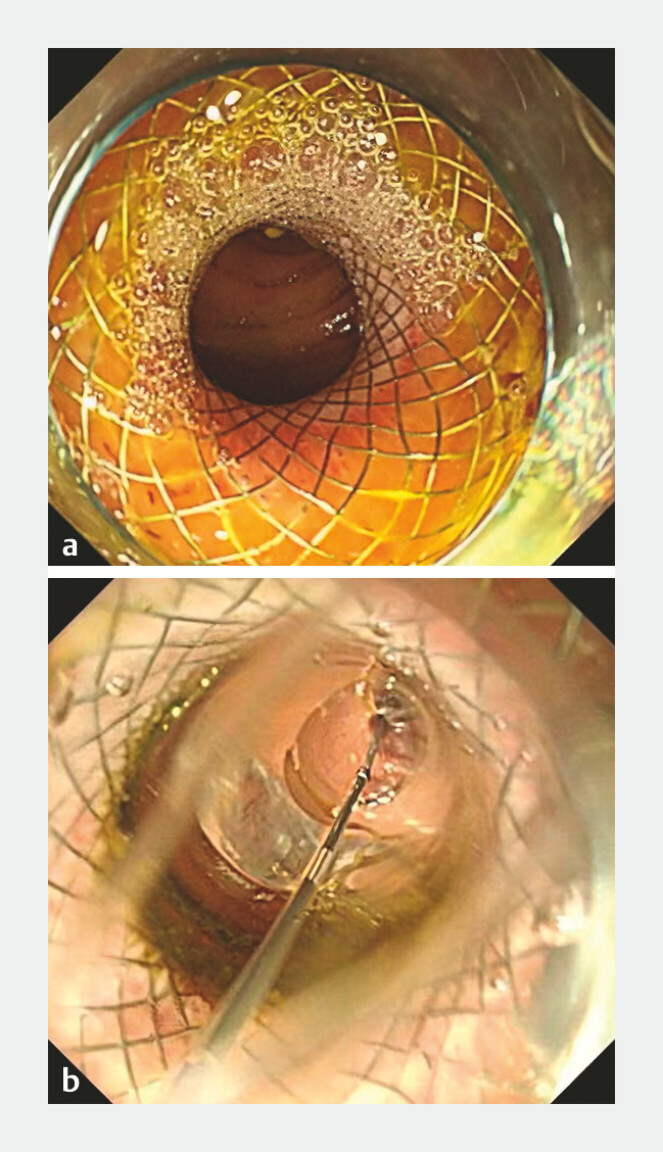
**a** Post-EUS-ICA imaging showing the patent anastomosis site. **b** Intraendoscopic fluoroscopy of anastomotic stoma balloon dilatation.

Malignant bowel obstruction (MBO) is a common complication in patients with advanced cancer, occurring in 10–28% of those with gastrointestinal malignancies. MBO can lead to dehydration, electrolyte imbalances, sepsis, intestinal perforation, and other serious complications that profoundly impair quality of life; the median survival ranges from 1 to 9 months. EUS-guided ileocolonic anastomosis is a safe and effective minimally invasive approach for palliation of MBO in high-risk patients with advanced cancer and severe comorbidities. It enables the rapid resolution of obstructive symptoms, restoration of bowel functions, and improvement in the quality of life, making it a valuable addition to the therapeutic armamentarium for MBO when surgical intervention is not feasible.

Endoscopy_UCTN_Code_TTT_1AS_2AZ

## Notes

Endoscopy E-Videos https://eref.thieme.de/e-videos
*E-Videos* is an open access online section of the journal *Endoscopy* , reporting on interesting cases and new techniques in gastroenterological endoscopy. All papers include a high-quality video and are published with a Creative Commons CC-BY license. Endoscopy E-Videos qualify for HINARI discounts and waivers and eligibility is automatically checked during the submission process. We grant 100% waivers to articles whose corresponding authors are based in Group A countries and 50% waivers to those who are based in Group B countries as classified by Research4Life (see: https://www.research4life.org/access/eligibility/ ). This section has its own submission website at https://mc.manuscriptcentral.com/e-videos .
